# Development of low-volume, high-intensity, aerobic-type interval training for elderly Japanese men: a feasibility study

**DOI:** 10.1186/s11556-017-0184-4

**Published:** 2017-08-24

**Authors:** Yosuke Osuka, Muneaki Matsubara, Ai Hamasaki, Yuji Hiramatsu, Hiroshi Ohshima, Kiyoji Tanaka

**Affiliations:** 1 Research Team for Promoting Independence of the Elderly, Tokyo Metropolitan Institute of Gerontology, Itabashi, Tokyo Japan; 2 Space Biomedical Research Group, Astronaut and Operation Control Unit, Japan Aerospace Exploration Agency, Tsukuba, Ibaraki Japan; 3 Faculty of Medicine, Department of Cardiovascular Surgery, University of Tsukuba, Tsukuba, Ibaraki Japan; 4 Graduate School of Comprehensive Human Sciences, University of Tsukuba, Tsukuba, Ibaraki Japan; 5 Faculty of Health and Sport Sciences, University of Tsukuba, Tsukuba, Ibaraki Japan

**Keywords:** High-intensity interval aerobic training, Elderly men, Feasibility

## Abstract

**Background:**

The purposes of this study were to identify 1) the feasibility of a novel exercise protocol (elderly Japanese male version of high-intensity interval aerobic training: EJ-HIAT) and 2) its preliminary data (%V̇O_2peak_, rating of perceived exertion) in comparison with traditional moderate-intensity continuous aerobic training (MICT).

**Results:**

Twenty-one sedentary elderly men, aged 60–69 years, performed two exercise protocols: EJ-HIAT, consisting of 3 sets of 2−3-min cycling at 75−85%V̇O_2peak_ with 1−﻿2-min active rests at 50%V̇O_2peak_ between sets, and MICT, consisting of 40-min cycling at 65%V̇O_2peak_. The completion rate, defined as the rate of participants who 1) did not demand withdrawal, 2) were not interrupted by the tester, and 3) did not change the workload during either exercise protocol, of EJ-HIAT was similar to that of MICT (EJ-HIAT: 100%, MICT: 95.2%). Maximal perceived exertion ratings assessed by Borg scale were also similar between EJ-HIAT and MICT. However, objectively measured maximal intensity assessed by %V̇O_2peak_ was higher for EJ-HIAT than for MICT (EJ-HIAT: 86.0 ± 5.6%, MICT: 67.1 ± 6.4%).

**Conclusion:**

These results suggested that EJ-HIAT has good feasibility and perceived exertion similar to MICT despite having higher objectively measured intensity than MICT. An intervention aimed as identifying the effects of EJ-HIAT on exercise tolerance should be performed in the future.

**Trial registration:**

UMIN000021185 (February 26, 2016).

## Background

Numerous studies have suggested that traditional moderate-intensity continuous aerobic training (MICT) improves exercise tolerance and reduces cardiovascular disease risk even in old age [1]. High-intensity interval training (HIIT), which has received substantial attention in several applied scientific fields [2, 3], including sports science [4], obesity prevention [5], cardiac rehabilitation [6, 7], and space medicine [8], is characterized by brief, intermittent bursts of vigorous activity interspersed with active rest periods [9]. Several meta-analyses have suggested that HIIT improves exercise tolerance more effectively than MICT [10–12].

We previously developed an original HIIT called the Japanese version of high-intensity interval aerobic training (J-HIAT) [13–17], which demonstrated greater improvement of exercise tolerance than MICT despite involving a lower volume and shorter duration of exercise, suggesting that J-HIAT could provide time-efficient training in sedentary people [17]. One consideration when developing the J-HIAT was whether astronauts could participate in exercise training safely, time-efficiently, and feasibly in the international space station [13, 14]. We believed that this process could be utilized to develop a more time-efficient exercise program to improve exercise tolerance in elderly people, based on the concept that countermeasures for declining exercise tolerance during space flight could be utilized to prevent declining exercise tolerance with aging.

The J-HIAT, consisting of 3 sets of 2−3-min cycling at vigorous intensity (first and second sets: 3 min at 85−90%V̇O_2peak_, third set: 3 min at 80−85%V̇O_2peak_) with 2-min active rests at 50%V̇O_2peak_ between each set, was developed for healthy, sedentary younger adults [17]. However, cross-sectional and longitudinal data have indicated that maximal oxygen consumption declines by approximately 10% per decade in healthy men [18, 19]. The J-HIAT was developed for younger adults and thus may be too difficult for elderly populations even if the workload is determined relatively. In this study, therefore, we aimed to identify 1) the feasibility of a novel elderly Japanese male version of the HIAT (EJ-HIAT) and 2) preliminary data (heart rate [HR], percentage of maximal oxygen consumption [%V̇O_2peak_], and rating of perceived exertion [RPE]) for comparison with traditional MICT.

## Methods

### Study procedures and participants

We conducted this study in accordance with the guidelines of the Declaration of Helsinki. The study protocol was approved by the Ethics Committee of the University of Tsukuba, Tsukuba University Hospital, and the Japan Aerospace Exploration Agency. This feasibility study was registered with the University Medical Information Network (UMIN000021185) in February 2016. Twenty-three elderly men, aged 60−69 years, were recruited from the southern area of Ibaraki through newspaper advertisements in March 2016 describing the inclusion criteria for the study: 1) no smoking history within 1 year, 2) not restricted by their doctor from participating in exercise, 3) not regularly participating in aerobic exercise at moderate intensity (assessed using the Borg RPE scale; <13, “somewhat hard”), 4) no participation in another clinical trial within 1 year, and 5) agreement to participate. All applications were received by telephone. After the study was explained, all participants provided written informed consent. Based on the results of a screening test (described below), participants were excluded by doctor who 1) had severe heart disease, cerebrovascular disease, or kidney disease, 2) were diagnosed electrocardiographically by a medical doctor with severe arrhythmia during rest and cardiopulmonary exercise (CPX), or 3) had knee joint pain during CPX.

### Screening test

After medical history taking, blood tests, electrocardiography, and CPX were performed to evaluate patients’ safety to perform exercise training. A nurse collected fasting blood samples from the antecubital vein of each participant. Total cholesterol, high-density lipoprotein cholesterol, low-density lipoprotein cholesterol, triglyceride, free fatty acid, urea nitrogen, creatinine, serum iron, white blood cell count, red blood cell count, hemoglobinometry, hematocrit, fasting blood glucose, Hb-A1c, immunoreactive insulin, homeostatic model assessment insulin resistance, and N-terminal pro-brain natriuretic peptide were measured and analyzed by Kotobiken Medical Laboratories (Ibaraki, Japan). Electrocardiography (ECG-1500, Nihon Kohden, Tokyo, Japan and DS-2150, Fukuda Denshi, Tokyo, Japan) was performed by a medical doctor to determine whether participants had severe arrhythmias during rest and CPX. CPX was performed to determine V̇O_2peak_. Details of the CPX measurement have been previously described [20]. Briefly, participants pedaled on a cycling ergometer (75XL III; Konami Sports Life, Tokyo, Japan) until physical exhaustion. After warming up for 2 min at 20 W, the workload increased after 1 min by 0.25 kp with 60 rpm of rotations. VO_2_ was measured using the breath-by-breath method with a computerized indirect calorimeter (Fitmate Pro, Cosmed, Rome, Italy) that can measure each 30-s average V̇O_2_. We used the value at the final 30-s interval as an indicator of V̇O_2peak_.

### Exercise protocols

**Fig. 1:**
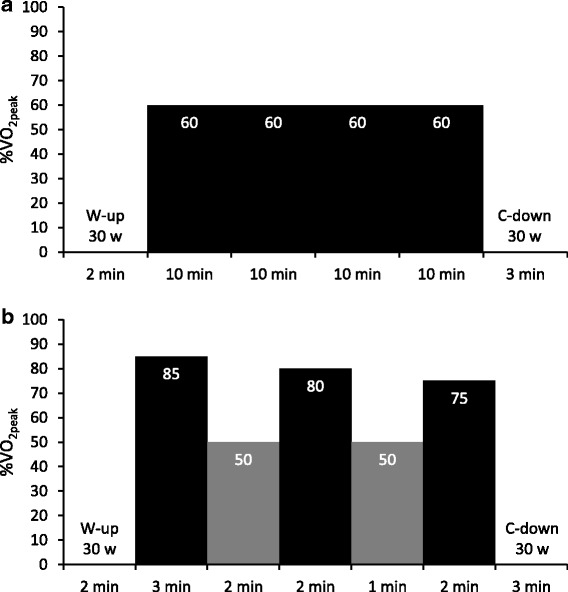
**a** EJ-HIAT and **b** MICT exercise protocols

To determine the appropriate intensity of MICT and EJ-HIAT for each participant, we calculated a simple linear regression equation using the values of V̇O_2_ (Y) and workload (X) per 30 s. First, %V̇O_2peak_ values were applied to the equation, and we calculated the intensity of both protocols for each participant [17]. In the first week, participants performed MICT; which consisted of 40-min cycling at 60% V̇O_2peak_ (Fig. 1a). This traditional exercise protocol has been recommended as a standard protocol for maintaining cardiovascular health in elderly people by the American College of Sports Medicine and American Heart Association [1]. The next week, participants performed the EJ-HIAT, consisting of 3 sets of 2−3-min cycling at vigorous intensity (first set: 3 min at 85%V̇O_2peak_, second set: 2 min at 80%V̇O_2peak_, third set: 2 min at 75%V̇O_2peak_) with 1−2-min active rests at 50%V̇O_2peak_ (first rest: 2 min, second rest: 1 min) between each set (Fig. 1b). The EJ-HIAT was amended specifically to be suited to elderly people. The EJ-HIAT has declining intensities at vigorous intensity (−5%V̇O_2peak_ at second set, −10%V̇O_2peak_ at third set), and decreased durations of vigorous intensity and active rest (−1 min during the second and third set of vigorous intensity and second set of active rest). These slight adjustments were performed depending on our heuristics. Consequently, the EJ-HIAT total duration was reduced by 3 min compared with the J-HIAT. The EJ-HIAT could be finished in 15 min: a 2-min warm-up, 10-min exercise, and 3-min cool-down. We decreased the actual workload by 10% from the calculated workloads for both protocols, since we anticipated that the participants would have difficulty performing these protocols without practice.

### Primary outcome measure

The primary outcome measure was the feasibility of each exercise protocol, assessed by completion rate. Completion was defined as participants 1) not demanding to withdraw, 2) not interrupted by the tester, and 3) who did not change the workload during either exercise protocol.

### Secondary outcome measures

Percent V̇O_2peak_, % HR_peak_, and RPE during the both exercise protocols were measured to assess their intensity. All data were collected from warm-up to cool-down. V̇O_2_ was measured every 30 s using the breath-by-breath method with a Fitmate Pro. HR and RPE data were collected at the last 15 s of each minute. HR was observed using electrocardiography (DS-2150, Fukuda Denshi, Tokyo, Japan). Overall body, breathing, and leg RPE were assessed using Borg’s RPE scale, ranging from 6−20 (6: no exertion, 11: light, 13: somewhat hard, 15: hard, 17: very hard, 20: maximal exertion).

### Statistical analysis

McNemar’s test and paired t tests were applied to compare differences in completion rate, average and peak point of V̇O_2_, HR, and RPE between the two exercise protocols. All analyses were performed using SPSS software (version 24.0, IBM Corp., Armonk, NY). *P* values of <0.05 were considered significant. All data are reported as n (%) or mean ± standard deviation.

## Results

### Study attrition

Before screening, one participant dropped out because of time constraints. After screening, one participant was excluded because of left anterior fascicular block arrhythmia. The other 21 participants performed both exercise protocols.

### Participant characteristics

Table 1 summarizes the characteristics of the study participants at screening. The participants’ average age was 67.6 ± 1.8 years. Twelve participants (57.1%) had mild diseases controlled by medication.

**Table 1:** Characteristics of study participants at the screening test (*n* = 21)

Age, years	67.6 ± 1.8
Height, cm	167.1 ± 6.6
Weight, kg	68.1 ± 8.7
Systolic blood pressure, mmHg	134.8 ± 15.4
Diastolic blood pressure, mmHg	84.0 ± 8.9
Resting heart rate, beats/min	70.3 ± 7.5
Hypertension, *n* (%)	7 (33.3)
Diabetes, *n* (%)	4 (19.0)
Hyperlipidemia, *n* (%)	2 (9.5)
Arthralgia, *n* (%)	10 (47.6)
Arrhythmia, *n* (%)	6 (28.6)
V̇O_2 peak_, mL/kg/min	27.8 ± 4.2
Heart rate peak, beats/min	154.9 ± 14.9
Albumin, g/dL	4.3 ± 0.2
Total cholesterol, mg/dL	210.0 ± 26.3
High-density lipoprotein cholesterol, mg/dL	63.6 ± 14.5
Low-density lipoprotein cholesterol, mg/dL	124.7 ± 20.6
Triglyceride, mg/dL	105.3 ± 41.6
Free fatty acid, mEq/L	0.7 ± 0.2
Urea nitrogen, mg/dL	15.8 ± 2.6
Creatinine, mg/dL	0.9 ± 0.1
Serum iron, μg	111.5 ± 43.0
White blood cell, μL	6166.7 ± 2050.4
Red blood cell, 10^4^μL	507.2 ± 48.3
Hemoglobinometry	15.5 ± 0.9
Hematocrit, %	47.8 ± 2.8
Fasting blood glucose, mg/dL	106.6 ± 18.3
Hb-A1c, %	6.1 ± 0.7
Immunoreactive insulin, μU/mL	6.4 ± 2.9
HOMA-R	1.7 ± 1.0
High-sensitivity C-reactive protein (>0.2 mg/dL), *n* (%)	2 (9.5)
NT-proBNP (>400 pg/mL), *n* (%)	0 (0)

*HOMA-R* homeostatic model assessment of insulin resistance, *NT-proBNP* N-terminal pro-brain natriuretic peptide)

### Adverse events and safety

No severe adverse events were observed during either exercise protocol, but transient asymptomatic tachycardia was observed in one participant during the EJ-HIAT cool-down. We quickly changed the workload to no-load and checked whole body symptoms whether hypotension and/or an anginal attack were initiated. Tachycardia was improved within 1 min, thereafter, did not relapse. This episode was appropriately assessed and treated by the medical doctor.

### Completion rate

One participant did not complete the MICT; he demanded a reduction in workload (completion rate, 95.2%). All participants completed the EJ-HIAT. Although the EJ-HIAT completion rate was higher, the protocols could not be compared statistically since the EJ-HIAT completion rate was 100%.

### Patterns of HR change

**Fig. 2:**
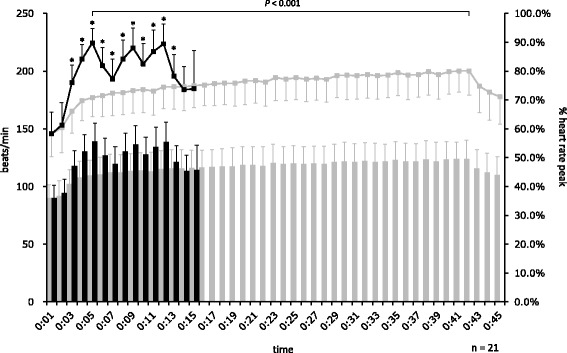
Comparison of HR between the EJ-HIAT and MICT protocols. Note: Line graph indicates the means and standard deviations of %HR_peak_. The bar graph indicates the means and standard deviations of HR. *: *P* < 0.05 (comparison with MICT)

The changes to HR are shown in Fig. 2. In the MICT, HR increased gradually over 40 min and peaked at 42:00 (HR: 123.8 ± 16.2 bpm, %HR_peak_: 80.1 ± 8.4%). In the EJ-HIAT, HR increased or decreased depending the workload, with the highest observed at 5:00 (HR: 139.1 ± 15.7 bpm, %HR_peak_: 89.8 ± 5.0%), 9:00 (HR: 136.3 ± 16.5 bpm, %HR_peak_: 84.1 ± 6.6%), and 12:00 (HR: 138.6 ± 17.3 bpm, %HR_peak_: 89.5 ± 6.9%). There were significant differences in HR between the protocols at each minute from 3:00−13:00 and in the peak point of HR at 42:00 in the MICT and at 5:00 in the EJ-HIAT.

### Oxygen uptake kinetics

**Fig. 3:**
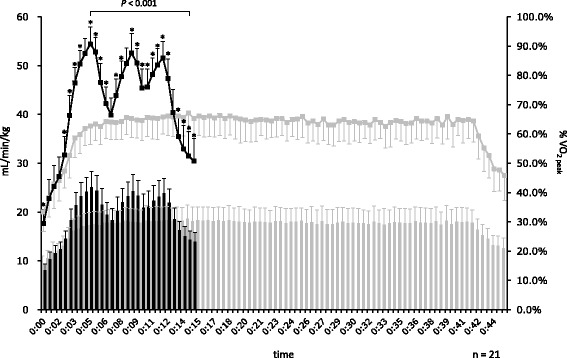
Comparison of V̇O2 between the EJ-HIAT and MICT protocols. Note: Line graph indicates the means and standard deviations of %V̇O_2peak_. The bar graph indicates the means and standard deviations of V̇O_2_. *: *P* < 0.05 (comparison with MICT)

V̇O_2_ kinetics are shown in Fig. 3. In the MICT, V̇O_2_ values increased gradually for the first 15 min, and then stabilized at approximately 17−18 ml/kg/min (63−65%V̇O_2peak_). The highest V̇O_2_ value occurred at 14:30 (V̇O_2_: 18.6 ± 2.8 ml/kg/min, %V̇O_2peak_: 67.1 ± 6.4%). In the EJ-HIAT, V̇O_2_ values increased or decreased depending on workload, with the highest observed at 5:00 (V̇O_2_: 25.1 ± 3.2 ml/kg/min, %V̇O_2peak_: 90.7 ± 5.8%), 9:00 (V̇O_2_: 24.3 ± 3.4 ml/kg/min, %V̇O_2peak_: 87.6 ± 6.7%), and 12:00 (V̇O_2_: 23.8 ± 3.1 ml/kg/min, %V̇O_2peak_: 86.0 ± 5.6%). V̇O_2_ significantly differed between the exercise protocols at 30-s intervals at 0:30, 2:30−6:30, 7:30−12:30, and 13:30−15:00. Peak V̇O_2_ value also significantly differed at 14:30 in the EJ-HIAT and at 5:00 in the MICT.

### Patterns of RPE change

**Fig. 4:**
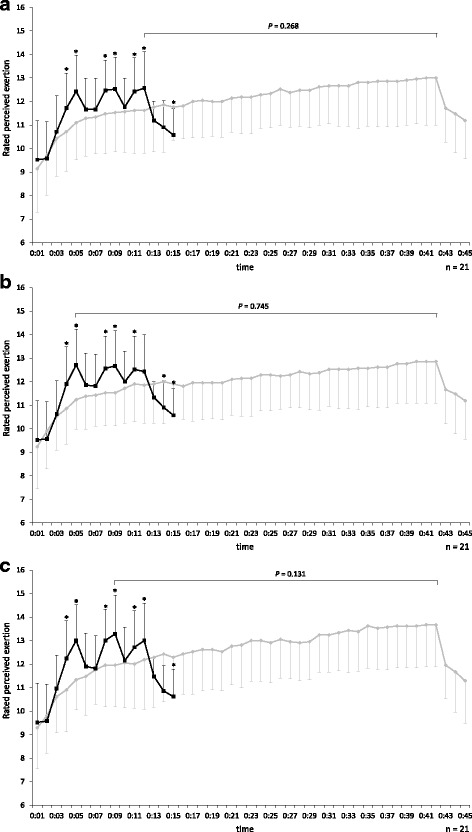
**a** :whole body, **b**: breathing, and **c**: legs. Comparison of RPE between the EJ-HIAT and MICT protocols. Note: Line graph indicates the means and standard deviations of RPE. *: *P* < 0.05 (comparison with MICT)

The change of RPE is shown in Fig. 4a, b, and c. In the MICT, whole-body, breathing, and leg RPE values increased gradually over 40 min, with the highest observed at 42:00 (whole body: 13.0 ± 2.0, breathing: 12.9 ± 1.8, legs: 13.7 ± 1.8). In the EJ-HIAT, the highest whole-body RPE values were observed at 5:00 (12.4 ± 1.5), 9:00 (12.5 ± 1.4), and 12:00 (12.6 ± 1.5). Whole-body RPE values at each minute significantly differed between the exercise protocols at 4:00−5:00, 8:00−9:00, 11:00−12:00, and 15:00. The highest breathing RPE values in the EJ-HIAT were observed at 5:00 (12.7 ± 1.5), 9:00 (12.7 ± 1.5), and 11:00 (12.5 ± 1.4). Breathing RPE values at each minute significantly differed between the exercise protocols at 4:00−5:00, 8:00−9:00, 11:00, and 14:00−15:00. The highest RPE values regarding legs in the EJ-HIAT were observed at 5:00 (13.0 ± 1.5), 9:00 (13.3 ± 1.6), and 12:00 (13.0 ± 1.6). Leg RPE values at each minute significantly differed between the exercise protocols at 4:00−5:00, 8:00−9:00, 11:00−12:00, and 15:00. There were no significant differences in peak RPE values between the exercise protocols.

## Discussion

This study is the first to report the feasibility of a novel HIIT protocol for elderly men (the EJ-HIAT) and provide preliminary data regarding HR, V̇O_2_, and RPE in comparison with traditional MICT. The EJ-HIAT had a similar completion rate and maximal perceived exertion to MICT, but greater objectively measured exercise intensity (V̇O_2_ and HR). These results suggest that the EJ-HIAT protocol has good feasibility and suitable intensity, warranting a future clinical intervention aimed to demonstrate its effects on exercise tolerance and the cardiovascular system.

### Feasibility of EJ-HIAT

Some previous studies, especially in European countries, have evaluated HIIT in elderly people. Wisloff et al. reported superior effects of walking-type HIIT on cardiovascular function-related outcomes compared to MICT and demonstrated that the HIIT was feasible even in elderly participants with chronic heart failure and impaired cardiovascular function; the participation rate of HIIT and MICT were 92 ± 2% and 95 ± 3%, respectively [6]. Iellamo et al. provided walking-type HIIT to patients with heart failure and observed 100% compliance to HIIT [21]. Haykowsky et al. reviewed 7 studies of HIIT in patients with heart failure and reduced ejection fraction and found similar completion rates between HIIT and MICT protocols (HIIT: 90%, MICT: 91%) [22]. These reports suggest that well-designed HIIT protocols have similar feasibility to MICT even in heart failure patients. Despite their good completion rates, these HIIT protocols have comparable durations to MICT (30−40 min). By contrast, the EJ-HIAT can be finished in 15 min, making it more feasible with regard to exercise duration. An intervention to assess the cardiovascular effects of EJ-HIAT is necessary in the future.

### Preliminary data

Our preliminary data indicate that the objective intensity of the EJ-HIAT as assessed by V̇O_2_ was temporarily but significantly higher than that of the MICT. Although we targeted vigorous intensity to 70−75%V̇O_2peak_, the actual data indicated that 86−90%V̇O_2peak_ was attained. Thus, actual intensity was approximately 15% higher than estimated. We may have underestimated V̇O_2peak_ at the screening, since participants were not used to the cycling test. Therefore, we will need to re-measure V̇O_2peak_ and adjust intensities accordingly during the intervention period in the future. Although objective intensity measurements (HR and V̇O_2_) were significantly higher, peak RPE values were not significantly different between the EJ-HIAT and MICT. RPE reflects not only physical exertion but also psychological exertion. Some participants gave opinions such as “MICT was boring,” indication that its duration was too long. These discomforts may negatively influence RPE. Bartlett et al. reported that ratings of perceived enjoyment were greater after undertaking HIIT compared with MICT, even if the RPE was higher for the HIIT than for MICT [23]. These results suggest that HIIT may be more enjoyable than MICT. Therefore, the HIIT may be advantageous with regard to participants’ enjoyment of vigorous activity, since the protocol is characterized by fluctuating intensity.

### Physiological and metabolic adaptations

Although the mechanisms regarding differences in physiological and metabolic adaptations between MICT and HIIT have yet to be definitively examined [9], exercise intensity may be a factor in the time-cost superiority of HIIT for those adaptations. Peroxisome proliferator-activated receptor γ coactivator (PGC)-1α is regarded as a key regulator of mitochondrial biogenesis in skeletal muscle [24], and exercise intensity was reported to influence its activation [25]. Burgomaster et al. reported that both MICT and HIIT increased peak oxygen uptake and total protein content of PGC-1α measured in muscle biopsy samples obtained before and after 6 weeks of training; nevertheless, total exercise volume was 90% lower in the HIIT [26]. In this way, physiological and metabolic adaptations during exercises after both training protocols would occur via the same pathway, but the time-cost influence of HIIT on those adaptations would be superior to those of MICT.

### Practical applications

Lack of time is one of the most common reasons that inhibits participation in exercise [27]. By contrast, the EJ-HIAT can be completed in only 15 min. Therefore, if the efficacy of the EJ-HIAT on cardiovascular system is validated by future trials, this protocol will provide a useful exercise program for older people who want to exercise efficiently.

### Limitations

This study has two limitations. First, it has a possibility of selection bias, since only elderly men participated in this study. Therefore, it is uncertain whether EJ-HIAT has suitable intensity in elderly women. Second, a blinded assessment was not conducted, so there is a possibility of observation bias in this study.

## Conclusion

Our preliminary data suggest that EJ-HIAT has feasibility comparable to MICT despite its significantly higher objectively measured intensity. An intervention aimed at identifying the effects of EJ-HIAT on exercise tolerance is needed in the future.

## Abbreviations

CPX: Cardiopulmonary exercise test
EJ-HIAT: Elderly Japanese male version of high-intensity interval aerobic training
HIIT: High-intensity interval training
HR: Heart rate
MICT: Moderate-intensity continuous aerobic training
RPE: Rating of perceived exertion
